# Violent crime, suicide, and premature mortality in patients with schizophrenia and related disorders: a 38-year total population study in Sweden

**DOI:** 10.1016/S2215-0366(14)70223-8

**Published:** 2014-06-01

**Authors:** Seena Fazel, Achim Wolf, Camilla Palm, Paul Lichtenstein

**Affiliations:** a Department of Psychiatry, University of Oxford, Warneford Hospital, Oxford, UK; b Department of Medical Epidemiology and Biostatistics, Karolinska Institutet, Stockholm, Sweden

## Abstract

**Background:**

People with schizophrenia and related disorders are at an increased risk of adverse outcomes, including conviction of a violent offence, suicide, and premature mortality. However, the rates of, and risk factors for, these outcomes need clarification as a basis for population-based and targeted interventions. We aimed to determine rates and risk factors for these outcomes, and investigate to what extent they are shared across outcomes and are specific to schizophrenia and related disorders.

**Methods:**

We undertook a total population cohort study in Sweden of 24 297 patients with schizophrenia and related disorders between January, 1972 and December, 2009. Patients were matched by age and sex to people from the general population (n=485 940) and also to unaffected sibling controls (n=26 357). First, we investigated rates of conviction of a violent offence, suicide, and premature mortality, with follow-up until conviction of a violent offence, emigration, death, or end of follow-up (Dec 31, 2009), whichever occurred first. Second, we analysed associations between these adverse outcomes and sociodemographic, individual, familial, and distal risk factors, for men and women separately, with Cox proportional hazards models. Finally, we assessed time trends in adverse outcomes between 1972 and 2009, for which we compared patients with unaffected siblings, and analysed associations with changes in the number of nights spent in inpatient beds in psychiatric facilities nationwide.

**Findings:**

Within 5 years of their initial diagnosis, 13·9% of men and 4·7% of women with schizophrenia and related disorders had a major adverse outcome (10·7% of men and 2·7% of women were convicted of a violent offence, and 3·3% of men and 2·0% of women died prematurely of any cause). During the study, the adjusted odds ratio of any adverse outcomes for patients compared with general population controls was 7·5 (95% CI 7·2–7·9) in men and 11·1 (10·2–12·1) in women. Three risk factors that were present before diagnosis were predictive of any adverse outcome: drug use disorders, criminality, and self-harm, which were also risk factors for these outcomes in unaffected siblings and in the general population. Over the period 1973–2009, the odds of these outcomes increased in patients with schizophrenia and related disorders compared with unaffected siblings.

**Interpretation:**

Schizophrenia and related disorders are associated with substantially increased rates of violent crime, suicide, and premature mortality. Risk factors for these three outcomes included both those specific to individuals with schizophrenia and related disorders, and those shared with the general population. Therefore, a combination of population-based and targeted strategies might be necessary to reduce the substantial rates of adverse outcomes in patients with schizophrenia and related disorders.

**Funding:**

Wellcome Trust and The Swedish Research Council.

## Introduction

People with schizophrenia have increased risks for a range of adverse outcomes, including violent outcomes,^1^ suicide,^2^ and premature mortality,^3^ compared with the general population. Odds of violent behaviour are reportedly up to seven-times higher in these patients than in the general population.^1^ For suicide, standardised mortality ratios are reportedly between 10 and 20, with a lifetime risk of 5%.^2^ High rates of overall mortality have also been reported,^3^, ^4^ although less is known about premature mortality. Research has focused on these outcomes separately, and rates and risk factors for any adverse outcome are rarely reported.^5^ Furthermore, little is known about the risk factors that lead to these outcomes, whether they are modifiable, and to what extent the factors are shared across the outcomes. Such information is necessary to trial therapies and preventive strategies to mitigate risks. Additionally, risk factor information is necessary to develop clinical prediction rules that would help in risk assessment.^6^

A related area of uncertainty and substantial debate is trends over time. In some studies, investigators have reported increased relative risks over time for suicide, death,^3^, ^7^ and convictions of a violent offence,^8^ but secular trends have made these data difficult to interpret.^9^ The emerging reinstitutionalisation of patients in some regions of the world^10^ might have been partly driven by concerns about deinstitutionalisation,^11^ although broader sociopolitical factors are probably important.^12^

To address these uncertainties, we have investigated all patients with diagnoses of schizophrenia and related disorders in secondary care in Sweden during a 38-year period to report the rates of specific and any serious adverse outcomes, clarify the contribution of some individual and parental risk factors, establish whether risk factors are shared between outcomes, and assess whether these determinants are similar to those in unaffected siblings and general population controls. If risk factors are similar to those in comparison groups, then universal population-based strategies to improve outcomes could be considered.^13^ Finally, we studied trends over time for the relative odds of each adverse outcome, and how these values varied with changes in inpatient bed numbers.

## Methods

### Study setting

We linked longitudinal nationwide Swedish population registers: the National Patient Register, the Medical Birth Register, and the Cause-of-Death Register (held at the National Board of Health and Welfare); the Longitudinal Integration Database for Health Insurance and Labour Market Studies and the Multi-generation Register (Statistics Sweden); and the Conscription Register (Swedish Defence Recruitment Agency). The Multi-generation Register connects every person born in Sweden in or after 1933, and ever registered as living in the country since 1960, to their parents.^14^ Similar information exists for immigrants who became citizens of Sweden before the age of 18 years, together with one or both parents. All residents, including immigrants, have a unique ten-digit personal identification number that is used in all national registers, which enables these data to be linked across these registers. We selected the cohort of people born between Jan 1, 1958, and Dec 31, 1994, who were followed from January, 1972 to the end of follow-up in December, 2009 (n=7 238 800).

We identified all 24 297 people who had been discharged from hospitals between 1972 and 2009 and who had been diagnosed with schizophrenia or a related disorder (other non-affective psychoses) on at least two separate occasions. We used the Multi-generation Register to identify unaffected siblings (n=26 357) of these patients. Every patient was linked to 20 age-matched and sex-matched controls. Their unaffected siblings were also independently linked to 20 age-matched and sex-matched controls. Both controls and siblings had no diagnoses of schizophrenia or related disorders, but might have had other psychiatric diagnoses.

### Measures

The National Patient Register includes data about all people admitted to any hospital for assessment, treatment, or both in Sweden (including secure hospitals and the few private providers of inpatient health care) from 1972 onwards, or people who had outpatient appointments with psychiatrists from 2001 onwards. Diagnoses are based on the International Classification of Diseases, eighth revision (ICD-8 [1972–86], code 295), ninth revision (ICD-9 [1987–96], codes 295; 297A, B, C, W, and X; and 298W and X), and tenth revision (ICD-10 [1997–2004], codes F20–F22 and F25–F29). We used two or more patient episodes as part of our inclusion criteria to increase diagnostic precision by minimising false-positive diagnoses.^15^ This definition includes any patients with two or more of the above diagnoses—that is, those with any two or more diagnoses of schizophrenia or other non-affective psychoses.

In terms of diagnostic validity, schizophrenia diagnoses in the Patient Register concord well with those obtained by an OPCRIT record review and interview (generating a Diagnostic and Statistical Manual of Mental Disorders, Fourth Edition [DSM-IV] diagnosis of schizophrenia), as shown by κ values of 0·74–0·76.^16^ In another study, 86% of hospital register schizophrenia diagnoses corresponded with diagnoses of DSM-IV schizophrenia syndrome made from file-based reviews by psychiatrists.^17^ However, the specificity is only fair at best.^16^ Therefore, some patients with a schizophrenia diagnosis in the Patient Register will be diagnosed with other mental disorders for any one inpatient episode, which led to our decision to use diagnoses on two different occasions to define cases.^18^ Less is known about comorbid psychiatric disorders, although one study showed fair to moderate agreement for comorbid substance use disorders in schizophrenia (κ=0·37, standard error 0·23, p<0·001, which corresponds to 68% full agreement).^19^ Since only about 1% of hospital admissions have missing personal identification numbers,^20^ the register has been used in a range of epidemiological investigations.^19^

For our outcome measure of premature mortality, we retrieved data about causes of death for all people who died between 1972 and 2009. We were mainly interested in rates of premature mortality, which we defined as death before the age of 56 years. We focused on premature mortality because prevention will make a substantial contribution to public health and to the present focus on population-based approaches to reduce mortality and morbidity.^21^ Deaths classified as suicide included undetermined deaths (ICD-10 codes Y10–Y34) because their exclusion would underestimate actual rates.^22^ Since the cause of death register covers more than 99% of deaths in Swedish residents, including those occurring outside Sweden, the loss of information about death by suicide was negligible.^23^ For our outcome measure of violent offences, we retrieved data for all convictions of a violent offence during the period 1972–2009 for all people in the cohort from the National Crime Register, which includes conviction data about all people 15 years of age (the age of criminal responsibility in Sweden) and older. Conviction of a violent offence was defined as homicide and attempted homicide, aggravated assault (an assault that is life-threatening in nature or causes severe bodily harm), common assault, robbery, arson, any sexual offence (rape, sexual coercion, child molestation, and sexual harassment [including indecent exposure]), and illegal threats or intimidation.^20^ We used conviction data because the criminal code in Sweden states that individuals are convicted as guilty irrespective of mental illness (ie, to be judged as not guilty by reason of insanity is not possible in Sweden). Thus, conviction data included people who received custodial or non-custodial sentences and those transferred to forensic hospitals (eg, people who were psychiatrically assessed and were thought to have suffered from psychosis or another severe mental disorder at the time of the offence). Furthermore, conviction data included cases in which the prosecutor decided to caution or fine the perpetrator (eg, less serious sexual crimes and some juvenile cases). Moreover, although some factors can affect sentencing, plea bargaining at the conviction stage is not part of the Swedish legal system.^24^ Therefore, conviction data accurately represent the extent of officially resolved criminality in the population. The crime register has complete national coverage—only 0·05% of all registered convictions had incomplete personal identification numbers in 1988–2000.^20^

For the sociodemographic risk factors that we assessed, we used family disposable income at the age of 15 years (divided into thirds) as a proxy for income, and we used this as a dichotomous variable (lowest tertile *vs* top two tertiles). If these data were unavailable, we used family disposable income at 16 years of age or at the youngest age at which it was available. Single marital status was defined as being unmarried at first diagnosis. Immigrant status was defined as being born outside of Sweden. We did not replace missing data by imputation or other methods.

The distal risk factors that we assessed were birth risk factors and intelligence quotient (IQ) scores. Birth risk factors were defined as at least one of the following: small for gestational age (2 or more SD below the mean);^25^ preterm birth (fewer than 37 completed weeks of gestation); or low birthweight (3·00–2·01 SD below mean birthweight).^26^

We extracted IQ scores (low, medium, and high) for Swedish men who underwent IQ testing at military conscription.^27^, ^28^ We standardised the global IQ score for both cases and controls against the whole population so that they followed a normal distribution between 1 and 9 with a mean of 5.^29^ In cases with schizophrenia and related disorders, IQ data were unavailable for 6001 (41%) men, which is higher than the missing levels in men in the control population (85 944/292 420; 29%); this is partly because people with psychiatric illness are exempt from conscription.

We defined drug and alcohol use disorders using inpatient (1972–2008) and outpatient (2001–08) primary or secondary diagnoses (ICD-8: 303, 304; ICD-9: 303, 304, 305·0, 305·9; ICD-10: F10–F19, except for x·5) in patients and controls. Alcohol and drug-related offences were defined as the illegal manufacture of alcohol or illegal drugs; driving when under the influence of alcohol or illicit substances; and the smuggling, manufacturing, possession or supply of illicit substances, and their personal use.

For parental risk factors, we extracted data for parental immigration status (born abroad), and suicide, convictions of a violent offence, psychiatric diagnoses, and alcohol or drug diagnoses, occurring before their child's first diagnosis of schizophrenia or a related disorder to avoid the possibility of reverse causality.

As a proxy for number of psychiatric inpatient beds, we extracted the total number of nights as an inpatient in a psychiatric hospital in the entire population of patients with psychiatric diagnoses for each year from 1972 to 2009.

### Statistical analysis

For between-population analyses, we estimated the association between a diagnosis of schizophrenia and causes of death, based on related work with matched controls,^19^, ^30^ for which we used the clogit command in Stata version 12.1. The clogit command fits conditional (fixed-effects) logistic regression models to matched case–control groups. We included two confounders (low family income and being born abroad) for theoretical reasons, and also tested whether each of these factors were independently associated with either case or control and outcome measures, respectively, in univariate analyses at the 5% significance level.^31^

For our sensitivity analyses, we also studied differences by diagnosis (schizophrenia *vs* other non-affective psychoses), for which we used only ICD-9 and ICD-10 diagnostic codes (since ICD-8 does not allow for differentiation between these diagnoses). In a further sensitivity analysis, we compared odds of adverse outcomes in the 1972–2000 cohort, for which only inpatient data were available, against the 2001–09 cohort, which had both inpatient and outpatient data. Finally, we stratified odds of adverse outcomes by age at diagnosis.

For within-population analyses, we followed patients with schizophrenia or related disorders longitudinally after diagnosis until their first conviction for a violent offence, suicide, emigration, death from any cause, or end of follow-up (Dec 31, 2009), whichever occurred first.

Our first set of analyses compared risks of outcomes in patients and unaffected siblings compared with general population controls matched for birth year and sex, in which we used conditional logistic regression to generate adjusted odds ratios. We then compared unaffected siblings with the general population to estimate the amount of familial confounding. A second set of analyses, that focused on risk factors, used Cox regression modelling that provided hazard ratios (HRs) with 95% CIs to account for time at risk. All analyses were stratified by sex. In adjusted Cox models, all risk factors significantly associated with the outcome variable after backward stepwise likelihood ratio regression were simultaneously taken into account. To assess the mechanisms behind the risk factors, we estimated hazard ratios, for the same outcomes and exposures, in unaffected siblings and controls from the general population.

For each outcome, we calculated odds ratios (adjusted for low income and marital status) by year of diagnosis in patients with schizophrenia and related disorders compared with age-matched and sex-matched controls, for which we used conditional logistic regression. We included age at first diagnosis (ie, age at start of follow-up) as an interaction term to account for period effects. Next, we similarly calculated odds ratios in unaffected siblings versus general population controls. Finally, we produced graphs to show annual ratios of these two odds ratios to compare relative trends in patients and unaffected siblings. Graphs show datapoints from 1979 onwards (since the incidence of sibling outcomes was low before this date), using 3-year rolling averages.

To test statistically for trends over time, we included the interaction term between casenesss and year of first diagnosis.^32^ The coefficient of this term shows the change in the odds ratio of an adverse outcome for each additional calendar year. In a separate trend analysis, we included the number of annual inpatient nights (in millions) as an interaction term to analyse the association between inpatient bed use and odds ratios of adverse outcomes.

The Regional Ethics Committee at the Karolinska Institutet approved the study (2009/939-31/5). Data were merged and anonymised by an independent government agency (Statistics Sweden), and the code linking the personal identification numbers to the new case numbers was destroyed immediately after merging. Therefore, informed consent was not required.

All analyses were done with SPSS, version 20.0 and Stata, version 12.1.

### Role of the funding source

The funders had no involvement in data collection, analysis, or interpretation; trial design; patient recruitment; any aspect pertinent to the study; or in the writing of the report or decision to submit for publication. SF had full access to all the data in the study and final responsibility for the decision to submit for publication.

## Results

Table 1 shows the descriptive statistics of 24 297 people in Sweden (14 261 men and 9676 women) with diagnoses of schizophrenia and related disorders between 1972 and 2009. The mean age at first diagnosis was 28·8 years (SD 7·8) for men and 29·8 years (8·3) for women (table 1). The mean follow-up time was 9·5 years (SD 7·6) for convictions of a violent offence, and 10·6 years (7·8) for mortality. Descriptive statistics for unaffected siblings (n=26 357) and general population controls (n=485 940) are presented in appendix pp 1–2.

**Table 1:** Descriptive data for risk factors in patients with diagnoses of schizophrenia and related disorders

		**Men (n=14 621)**	**Women (n=9676)**
Schizophrenia		5704 (39·0%)	2846 (29·4%)
Related disorders (excluding schizophrenia)		8917 (61·0%)	6830 (70·6%)
Socioeconomic factors			
	Mean age at first diagnosis, years (SD)	28·8 (7·8)	29·8 (8·3)
	Income in lowest tertile	5723 (39·1%)	4026 (41·8%)
	Born abroad	3639 (24·9%)	2400 (24·8%)
	Single	11 592 (79·3%)	6588 (68·1%)
Parental factors before diagnosis			
	Alcohol or drug use disorders	809 (5·5%)	463 (4·8%)
	Any offence	911 (6·2%)	594 (6·1%)
	Violent offence	423 (2·9%)	266 (2·7%)
	Suicide	268 (1·8%)	202 (2·1%)
	Psychiatric diagnosis	1369 (9·4%)	872 (9·0%)
	Born abroad	1930 (13·2%)	1108 (11·5%)
Individual factors before diagnosis			
	Alcohol use disorders	1047 (7·2%)	410 (4·2%)
	Drug use disorders	1501 (10·3%)	600 (6·2%)
	Alcohol offence	1736 (11·9%)	169 (1·7%)
	Drug offence	2156 (14·7%)	351 (3·6%)
	Alcohol or drug medication	198 (1·4%)	64 (0·7%)
	Any offence	6507 (44·5%)	1639 (16·9%)
	Violent offence	3409 (23·3%)	497 (5·1%)
	Non-violent offence	5966 (40·8%)	1501 (15·5%)
	Self-harm	1419 (9·7%)	1440 (14·9%)
IQ*			
	High (7–9)	1123 (13·0%)	NA
	Medium (4–6)	3987 (46·3%)	NA
	Low (1–3)	3510 (40·7%)	NA
Birth complications†		411 (11·9%)	240 (11·7%)

Data are n (%), unless otherwise indicated. IQ=intelligence quotient. NA=not available.

* IQ data were available for 8620 men.

† Data for birth complications were available for a sample of 3445 men and 2058 women.

**Figure 1:**
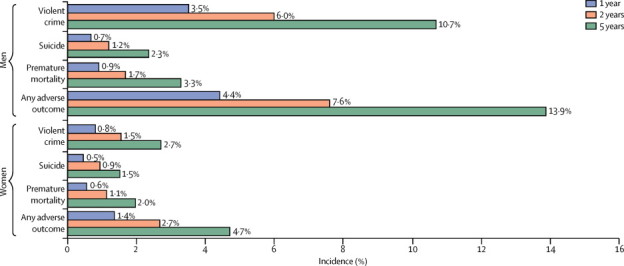
Incidence of conviction of a violent offence, suicide, premature mortality, and any adverse outcome within 1, 2, and 5 years of first diagnosis in patients with schizophrenia and related disorders

Of the diagnosed patients, 13·9% of men and 4·7% of women had a major adverse outcome within 5 years of first diagnosis (figure 1). During the study period, the adjusted odds ratio of any adverse outcomes for patients compared with the general population controls was 7·5 (95% CI 7·2–7·9) in men and 11·1 (10·2–12·1) in women. Odds of convictions for a violent offence, suicide, and premature mortality rates were higher in patients with schizophrenia and related disorders than in the general population, with some evidence of familial confounding shown by the results in the unaffected siblings group (table 2).

**Table 2:** Rates and adjusted odds ratios of adverse outcomes in patients with schizophrenia and related disorders, in unaffected siblings, and in general population controls

		**Patients with schizophrenia and related disorders (n=24 297)**	**Unaffected siblings (n=26 357)**	**General population (n=485 940)**	**Patients *vs* general population, aOR (95% CI)**	**Siblings *vs* general population, aOR (95% CI)**	**Patient *vs* sibling analyses, ROR (95% CI)**
Men, n (%)		14 621 (60·2%)	13 577 (51·5%)	292 420 (60·2%)	NA	NA	NA
Women, n (%)		9676 (39·8%)	12 780 (48·5%)	193 520 (39·8%)	NA	NA	NA
Violent offence (per 10 000 person-years)		2958 (129·1)	1005 (26·5)	9284 (19·5)	7·4 (7·1–7·8)	1·8 (1·7–1·9)	4·2 (3·8–4·5)
	Men	2431 (184·1)	869 (46·1)	8590 (29·9)	6·6 (6·3–6·9)	1·7 (1·6–1·9)	3·8 (3·5–4·2)
	Women	527 (54·3)	136 (7·2)	704 (3·7)	14·9 (13·2–16·8)	2·2 (1·9–2·7)	6·7 (5·4–8·3)
Premature mortality (per 10 000 person-years)		1593 (80·0)	631 (16·1)	4171 (11·0)	8·1 (7·6–8·6)	1·6 (1·5–1·7)	5·1 (4·6–5·7)
	Men	1126 (102·4)	427 (21·4)	3005 (14·1)	8·1 (7·5–8·7)	1·7 (1·5–1·8)	4·8 (4·2–5·4)
	Women	467 (45·8)	204 (10·6)	1166 (6·2)	8·1 (7·2–9·1)	1·4 (1·2–1·7)	5·6 (4·7–6·8)
Suicide (per 10 000 person-years)		889 (34·5)	180 (4·6)	866 (1·8)	20·7 (18·8–22·9)	2·6 (2·2–3·1)	7·9 (6·6–9·6)
	Men	625 (40·2)	124 (6·2)	703 (2·4)	18·3 (16·3–20·5)	2·5 (2·0–3·0)	4·9 (4·3–5·5)
	Women	264 (25·9)	56 (2·9)	163 (0·9)	31·1 (25·3–38·2)	3·0 (2·2–4·0)	10·5 (7·3–15·0)

Data in the first three columns are n (rate per 10 000 person-years), unless otherwise indicated. General population controls are matched by age and sex. aOR=adjusted odds ratios (adjusted for low family income and being born abroad). ROR=ratio of odds ratios.

In people with schizophrenia and related disorders, 10·7% of men and 2·7% of women were convicted of a violent offence within 5 years of their first diagnosis (figure 1). Some sociodemographic factors (low family income and being born abroad), and individual factors before first diagnosis (previous conviction for a violent offence, drug and alcohol use disorders, and in men, self-harm) were associated with violent convictions in patients (table 3) and in unaffected siblings (table 4). Low IQ was a risk factor in men (HR [adjusted for age] 1·8, 95% CI 1·5–2·2, for a comparison between low and high IQ; appendix pp 3–4). Birth complications, being single, and parental suicide were not associated with violent convictions in patients with schizophrenia and related disorders (appendix pp 3–4). Risk factors in the comparison groups followed the same trend as in the patients, but hazard ratios tended to be higher in unaffected siblings and in the general population than in the patients (see appendix pp 3–4 for univariate associations).

**Table 3:** Multivariate risk factor models for conviction of a violent offence, suicide, and premature mortality in patients with schizophrenia and related disorders (n=24 297)

	**Violence**		**Suicide**		**Premature mortality**	
	HR (95% CI)	p value	HR (95% CI)	p value	HR (95% CI)	p value
**Men**						
Low family income	1·2 (1·1–1·3)	<0·0001	..	..	..	..
Born abroad	1·5 (1·4–1·7)	<0·0001	0·7 (0·6–0·9)	0·0014	0·8 (0·7–1·0)	0·0127
Single status	..	..	0·6 (0·5–0·7)	<0·0001	0·7 (0·6–0·8)	<0·0001
Parental alcohol/drug use disorders	1·3 (1·1–1·5)	0·0044	..	..	1·3 (1·0–1·7)	0·0247
Parental violent offence	1·4 (1·2–1·8)	0·0011	0·4 (0·2–0·8)	0·0121	0·3 (0·1–0·5)	0·0002
Parental suicide	..	..	1·6 (1·1–2·6)	0·0298	..	..
Alcohol use disorders	1·4 (1·2–1·6)	<0·0001	..	..	1·4 (1·1–1·7)	0·0053
Drug use disorders	1·8 (1·6–2·0)	<0·0001	1·3 (1·0–1·7)	0·0327	1·5 (1·2–1·8)	0·0001
Previous violent offence	3·4 (3·1–3·7)	<0·0001	1·4 (1·1–1·7)	0·0009	1·4 (1·2–1·6)	<0·0001
Self-harm	1·3 (1·1–1·4)	0·0004	2·0 (1·6–2·4)	<0·0001	1·6 (1·3–1·9)	<0·0001
**Women**						
Low family income	1·3 (1·1–1·6)	0·0031	..	..	1·3 (1·1–1·6)	0·0075
Born abroad	1·5 (1·2–1·6)	0·0002	..	..	..	..
Single status	..	..	0·7 (0·5–0·9)	0·0098	..	..
Parental alcohol/drug use disorders	..	..	..	..	..	..
Parental violent offence	..	..	..	..	..	..
Parental suicide	..	..	..	..	..	..
Alcohol use disorders	2·1 (1·5–2·9)	<0·0001	1·8 (1·1–3·0)	0·0149	1·6 (1·1–2·3)	0·0241
Drug use disorders	2·8 (2·1–3·6)	<0·0001	..	..	1·9 (1·4–2·6)	0·0001
Previous violent offence	5·1 (4·0–6·5)	<0·0001	..	..	..	..
Self-harm	..	..	2·3 (1·7–3·0)	<0·0001	1·9 (1·5-2·4)	<0·0001

HR=hazard ratio. HRs are additionally adjusted for age at diagnosis.

**Table 4:** Multivariate risk factor models for conviction of a violent offence, suicide, and premature mortality in unaffected siblings (n=26 357)

	**Violence**		**Suicide**		**Premature mortality**	
	HR (95% CI)	p value	HR (95% CI)	p value	HR (95% CI)	p value
**Men**						
Low family income	1·3 (1·2–1·5)	0·0001	..	..	..	..
Born abroad	1·7 (1·5–2·1)	<0·0001	..	..	..	..
Parental alcohol/drug use disorders	..	..	..	..	..	..
Parental violent offence	1·9 (1·4–2·7)	0·0002	..	..	..	..
Parental suicide	1·6 (1·1–2·3)	0·0245	..	..	..	..
Alcohol use disorders	2·3 (1·8–3·0)	<0·0001	2·5 (1·4–4·7)	0·0032	2·5 (1·7–3·5)	<0·0001
Drug use disorders	2·4 (1·8–3·1)	<0·0001	7·6 (4·1–14·1)	<0·0001	4·5 (3·1–6·5)	<0·0001
Previous violent offence	7·2 (6·2–8·3)	<0·0001	1·7 (1·1–2·9)	0·0253	2·3 (1·8–3·0)	<0·0001
Self-harm	1·9 (1·4–2·6)	<0·0001	2·5 (1·3–4·8)	0·0073	1·8 (1·2–2·7)	0·0072
**Women**						
Low family income	1·7 (1·2–2·5)	0·0020	..	..	..	..
Born abroad	..	..	..	..	..	..
Parental alcohol/drug use disorders	..	..	..	..	..	..
Parental violent offence	..	..	..	..	..	..
Parental suicide	..	..	..	..	..	..
Alcohol use disorders	3·6 (1·8–7·0)	0·0002	..	..	3·8 (1·8–7·8)	0·0003
Drug use disorders	3·6 (1·8–7·3)	0·0003	..	..	..	..
Previous violent offence	5·9 (3·2–11·0)	<0·0001	..	..	..	..
Self-harm	3·5 (2·0–6·2)	<0·0001	10·2 (5·4–19·4)	<0·0001	4·1 (2·5–6·7)	<0·0001

HR=hazard ratio. HRs are additionally adjusted for age at diagnosis.

2·3% of men and 1·5% of women with schizophrenia and related disorders died by suicide within 5 years of their first diagnosis (figure 1). In men, not being single at the time of diagnosis, being born in Sweden, parental suicide before diagnosis, and three individual risk factors (previous conviction for violence, drug use disorders, and self-harm) were associated with suicide (table 3). In women, not being single, self-harm, and alcohol use disorders were risk factors for suicide (table 3). In men, parental conviction of a violent offence was inversely associated with suicide, and low IQ was associated with reduced risk of suicide (adjusted odds ratio 0·8, 95% CI 0·6–1·0; appendix pp 5–6). In univariate analyses, we noted differences in the direction of effects in risk factors in the unaffected sibling and general population controls. Low income and low IQ were associated with suicide in both the sibling and general population control groups, whereas they were not in patients with schizophrenia and related disorders (appendix pp 5–6). In multivariate models in unaffected siblings, suicide was associated with drug and alcohol use disorders and previous conviction of a violent offence in men, and with self-harm in both sexes (table 4).

The odds of premature mortality were substantially higher in patients with schizophrenia and related disorders than in unaffected siblings and general population controls (table 2). There was substantial overlap with suicide risk factors, since half of all deaths in the study were deaths by suicide. One difference was that, in men, birth complications were associated with mortality (HR 1·9, 95% CI 1·2–2·9; appendix pp 7–8) but not suicide. Indicators of drug and alcohol misuse were significant in multivariate analyses (table 3), in unaffected siblings (table 4), and in solely non-suicide deaths (data not shown).

In our sensitivity analyses, we noted no significant differences in odds of adverse outcomes between patients with schizophrenia and related disorders, or between those diagnosed before 2001 and those diagnosed since then. Odds of conviction of a violent offence were highest in people diagnosed between 25 and 44 years of age (appendix p 9).

**Figure 2:**
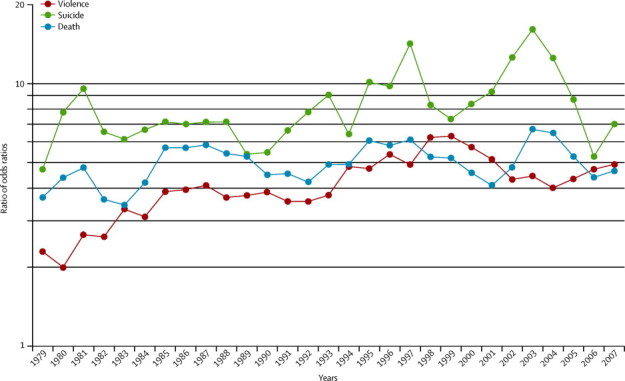
Ratio of odds ratios of adverse outcomes, by year of diagnosis The graph presents 3-year rolling averages of the ratios of odds ratios of adverse outcomes (comparison of analyses of patients with schizophrenia and related disorders *vs* unaffected siblings). In statistical analyses, the ratio of odds ratios for conviction of a violent offence increased significantly by 1·1% (95% CI 0·1–2·2%) per year. This ratio of odds ratios also increased, but not significantly, for suicide (1·7% increase per year [95% CI −0·9 to 4·3]) and did not change significantly for premature mortality (0·2% increase per year [−1·3% to 1·6%]).

We recorded evidence of an increase between 1972 and 2009 in the adjusted odds ratios of violence, suicide, and premature mortality in patients with schizophrenia and related disorders compared with general population controls (table 5). To test for residual confounding in this association, we also investigated trends for risks of the adverse outcomes in unaffected siblings compared with general population controls, and noted an increase in the odds ratio of premature mortality, but not violence or suicide. Finally, in a comparison of these two analyses (and thus comparing patients with their unaffected siblings), we noted a signifcant average yearly increase in the ratio of odds ratios of violence of 1·1% (95% CI 0·1–2·2%; figure 2). This ratio of odds ratios increased, but not significantly, for suicide (1·7% increase per year, 95% CI −0·9% to 4·3%), and did not change significantly for premature mortality (0·2% increase by year, 95% CI −1·3 to 1·6; figure 2).

**Table 5:** Effects of year of diagnosis and inpatient nights per year on adjusted odds ratios of adverse outcomes

	**Patients *vs* general population analysis, % change in aOR (95% CI)**	**Unaffected siblings *vs* general population analysis, % change in aOR (95% CI)**	Ratio of odds ratios*
**% increases in odds ratios, by calendar year of diagnosis**			
Violence	1·3% (0·6–2·0)	0·1% (−0·7 to 1·0)	1·1% (0·1 to 2·2)
Suicide	2·6% (1·0–4·3)	1·0% (−1·0 to 3·0)	1·7% (−0·9 to 4·3)
Premature mortality	1·6% (0·5–2·7)	1·4% (0·4 to 2·5)	0·2% (−1·3 to 1·6)
**% increases in odds ratios, by 1 000 000 fewer inpatient nights per year**			
Violence	5·6% (3·5–7·6)	−0·4% (−2·3 to 2·1)	5·6% (2·6 to 8·4)
Suicide	7·7% (3·3–11·8)	2·3% (−2·9 to 7·2)	5·5% (−1·3 to 11·8)
Premature mortality	5·1% (2·3–7·8)	1·7% (−1·0 to 4·4)	3·4% (−0·6 to 7·2)

aOR=adjusted odds ratios (adjusted for low family income and being born abroad; controls matched by age and sex).

* The ratio of odds ratios measures the changes in rates of adverse outcomes in patients compared with unaffected siblings and is the ratio of the values in the first two columns.

The number of inpatient nights per year decreased steadily by an average of 0·3 million nights annually between 1973 and 2009 (data not shown). In an analysis of the relation between inpatient nights per year and adverse outcomes, we showed that the number of inpatient nights was negatively associated with violence but had no significant associations with suicide or premature mortality (table 5)—that is, fewer annual inpatient nights were associated with more violence perpetrated by those with schizophrenia and related disorders compared with unaffected siblings.

Additional adjustments for substance use disorders had little effect on our findings (appendix p 10).

## Discussion

In this study of 24 297 people with schizophrenia and related disorders who were followed up for 38 years, we analysed rates and risk factors for three serious adverse outcomes. Within 5 years of first diagnosis, 10·7% of men with schizophrenia and related disorders were convicted of a violent offence, 2·3% died from suicide, and 3·3% died from all causes. In women, within 5 years of first diagnosis, 2·7% were convicted of a violent offence, 1·5% died from suicide, and 2·0% died from all causes. Overall, 13·9% of the men and 4·7% of the women had one of these outcomes within 5 years of their diagnosis. Although substantial research has focused on primary prevention in schizophrenia, our data suggest that secondary prevention of adverse outcomes remains a major challenge.

Investigation of risk factors for these outcomes should help to provide targets for intervention. We assessed a range of sociodemographic, parental, and individual risk factors, and four main findings emerged. First, three risk factors—drug use disorders, a history of violent criminality, and self-harm—typically increase the risks of all three outcomes, and are all present before diagnosis. This finding could allow the identification of high-risk patients, but also suggests that those people at high risk of developing psychosis could benefit from interventions that reduce drug use, self-harm, and criminality. Early intervention services, which are increasingly being trialled in many countries,^33^ could prioritise such interventions, in addition to those that reduce the likelihood of progression to schizophrenia.

The parallel finding—that many risk factors were not shared across different outcomes—suggests that risk assessment and management might have to be carefully tailored for specific outcomes.^34^ Thus, the development of clinical prediction methods for more than one adverse outcome might have low accuracy and discriminatory ability. Additionally, it suggests that mechanisms could be different for violence and suicide in schizophrenia. Alcohol use disorders, low IQ, and parental conviction were risk factors for conviction of a violent offence but not suicide, and being born abroad was protective for suicide risk, but not for violent conviction, in patients with schizophrenia and related disorders. We reported new findings for two distal risk factors—birth complications, which have been shown to be related to violence in general population samples,^35^ and IQ, for which previous research has found an inverse relation with suicide in patients with schizophrenia.^36^, ^37^ For birth complications, associations were at most modest, partly because they are related directly to the disease cause.^38^

Our study of risk factors contrasts with previous systematic reviews. Unlike a recent systematic review of violence risk factors,^39^ we recorded associations with self-harm, IQ, and parental conviction of a violent offence. For suicides, by contrast with a previous review,^36^ we noted that conviction of a violent offence and not being single increased the risk of suicide. Furthermore, we assessed a range of parental and criminality factors not included in the suicide review. The advantage of incorporating premature mortality is that it might include deaths that have been misclassified and attributable to external causes apart from suicide (eg, accidents and homicides). Previous work on premature mortality has mainly focused on physical characteristics and diseases.^40^ Therefore, to our knowledge, our findings about alcohol and drug use, self-harm, birth complications, and previous conviction as risk factors for premature mortality, and non-suicide deaths specifically, are novel (panel).

Panel **Research in context**

**Systematic review**

We searched Medline from 1946 up until Feb 10, 2014, with no language or date restrictions, for articles about patients with schizophrenia or related disorders that used genetically informed designs, and assessed adverse outcomes (violence, suicide, and premature mortality), associated risk factors, and time trends. The following search terms were used: (“schizo*” or “psychos*” or “psychot*”) and (“sibling*” or “twin*”) and (“mortality” or “suicid*” or “viol*” or “homicide” or “crim*”). We identified three primary studies: one sibling comparison study into violence (n=8003),^19^ a twin comparison investigation about criminality (n=280),^41^ and one twin report on mortality (n=590).^42^ Furthermore, we searched Medline specifically for systematic reviews (with use of the same search terms for disorder and outcome, but without “sibling” or “twin”) and identified five reviews about adverse outcomes in schizophrenia, specifically on comparative rates of violence^1^ and mortality,^3^ and risk factors for violence,^39^ suicide,^2^, ^36^ and mortality.^40^ Additionally, we found two reviews that report on the risk of re-offending.^43^, ^44^ We did not find any systematic reviews or sibling designs that assessed time trends.

**Interpretation**

Previous studies showed patients with schizophrenia to be at higher risk of committing violent offences (risk ratio 1·8)^41^ and of premature death (standardised mortality ratio 1·8)^42^ than unaffected twins. Through the use of a sibling control design to account for familial (genetic and early environmental) confounding, our study of 24 297 patients with schizophrenia and related disorders substantially increases the amount of available evidence about adverse outcomes in patients with these disorders, and includes more than two-times more cases than previous studies combined. We have reported that schizophrenia and related disorders are associated with substantially increased rates of adverse outcomes. Our risk estimate for violent crime is higher than that in a previous study,^19^ which is partly explained by the present study including violent crime from first diagnosis in the cases and from the same time in the control participants. Furthermore, we have used the broader diagnostic category of schizophrenia and related disorders, whereas the previous report studied only schizophrenia.^19^ By contrast with previous population-based studies,^1^ we have included both outpatients and inpatients. Drug misuse, previous criminality, and self-harm, all identified before the first diagnosis of schizophrenia and related disorders, were risk factors for the three adverse outcomes investigated (violent crime, suicide, and premature mortality). These same factors increased the risk of these outcomes in unaffected siblings and in the general population.^45^, ^46^ This finding suggests that a combination of both population-based and targeted strategies for patients with schizophrenia and related disorders could reduce the substantially raised risks of violence, suicide, and premature mortality in these people.

A fourth aim of this study was to analyse these risk factors in unaffected siblings and in general population controls, which is, to our knowledge, the first time that this has been done. The inclusion of unaffected siblings allows familial (genetic and/or early environmental) confounding to be accounted for, and hence any differences would be specific to people developing the illness. Since most risk factors were similar in patients and their unaffected siblings, we conclude that no clear familial confounding exists for the effects of these determinants. Thus, even if the development of schizophrenia is affected by a genetic liability, risk factor prevention and management is likely to lead to a reduction in adverse outcomes. This approach is informative as to whether population-based strategies can reduce adverse outcomes in patients and in the general population. We have shown that risk factors were mostly similar in patients and both comparison groups, including those for drug use disorders, criminality, and self-harm. Therefore, population-based strategies to reduce these risk factors could be prioritised as one public health approach to reduce violence and premature mortality.

However, we noted two exceptions to this finding, both of which were for suicide outcomes. Low IQ was a risk factor for suicide in the general population and in unaffected siblings but protective (albeit non-significantly) in patients with schizophrenia and related disorders. The latter finding has been reported in all patients with psychiatric disorders^47^ and in those with schizophrenia,^36^, ^37^ and is thought to be mediated by patients with higher intelligence having greater insight into the chronicity of the illness. The other finding, which to our knowledge has not been reported elsewhere, is that parental conviction of a violent offence was inversely related to suicide in patients but increased the suicide risk in the general population. Furthermore, we did not record an association in the siblings, suggesting that the inverse relation in patients was confounded by either genetic factors or early environmental ones. A possible explanation is that people with genetic predispositions to violence are more likely to externalise violence than to internalise it.

Trends in adverse outcomes in the past few decades have caused substantial controversy in psychiatry.^11^ Similar to many European countries, Sweden experienced gradual de-institutionalisation from the 1970s. We used a new approach to study trends, and by comparing risk of adverse outcomes in patients versus their unaffected siblings, we have tried to adjust for residual confounding; in other words, we have attempted to take account of secular changes that would differentially affect the families of patients with severe mental illness compared with the general population (eg, income or drug and alcohol use that does not come to medical attention). Additionally, by investigating the broad diagnostic category of schizophrenia and related disorders, we limited the effects of changes over time in classification between individual psychoses. For conviction of a violent offence, the risk over time increased compared with both general population controls and unaffected siblings. This trend was also associated with changes in inpatient bed numbers. For suicide and premature mortality, over time risks increased in the patients compared with general population controls, and these risks remained raised in sibling comparisons, although not significantly: probably because of few outcomes in unaffected siblings. Previous studies have used general population comparisons and reported significantly increased premature mortality,^3^, ^7^, ^48^ and suicide^49^ over time, and our study confirms these findings with a more robust methodology. None of the trends that we studied were downward trends, which suggests that new treatments have not reduced adverse outcomes for these patients in Sweden.

Our study benefits from several strengths: the use of population registers and at least two patient episodes allowed us to obtain good diagnostic specificity with a large sample size. Furthermore, through register linkage, we analysed risk factors occurring before diagnosis, and risk factors in the general population and in unaffected sibling controls. However, the limitations of our study include a restricted set of risk factor information and the fact that the study was done in one country. However, the prevalence of schizophrenia,^48^ violent assault,^50^ and suicide^51^ in Sweden are similar to those in other high-income countries. Generalisability to other high-income countries is also supported by an internationally recognised measure of psychiatric morbidity—age-adjusted disability-adjusted life-years—which is similar in Sweden and other high-income countries.^52^ Prevalences of psychotic disorders hardly differ across European countries, with a median prevalence of 1·0% for psychotic illness.^53^ Another limitation is that we used patient registers, and therefore the prevalences of drug and alcohol use disorders will have been underestimated, especially in population controls who might not come into contact with health services. However, this sample represents individuals who access services and, therefore, those to whom interventions could potentially be provided. Reliance on inpatients before 2001 might have overestimated risks of adverse outcomes, although the risk estimates did not differ in the cohort after 2001 when outpatient data were included. We did not investigate modification of risk factors through treatment because these data have recently become available and solely for medication. Finally, the control participants in our sample might include people with other mental health problems but no diagnoses of schizophrenia and related disorders. Therefore our findings might be conservative estimates, since other mental disorders are associated with adverse outcomes.

In conclusion, schizophrenia and related disorders are associated with substantially increased rates of convictions of a violent offence, suicide, and premature mortality. We have shown that three risk factors are associated with these outcomes, which are the same as the factors that increase the risk of these outcomes in unaffected siblings and in the general population. A combination of population-based and targeted strategies might be necessary to reduce the risks of violence, suicide, and premature mortality in patients with schizophrenia and related disorders.
