# Supplementary material for: Violent crime, suicide, and premature mortality in patients with schizophrenia and related disorders: a 38-year total population study in Sweden
Source: Lancet Psychiatry. 2014 Jun 1;1(1):44–54. doi: 10.1016/S2215-0366(14)70223-8 (PMC4124855; doi:10.1016/S2215-0366(14)70223-8)
Supplement: Supplementary appendix [file mmc1.pdf]

THE LANCET Psychiatry

Supplementary appendix

This appendix formed part of the original submission and has been peer reviewed. We post it as supplied by the authors.

Supplement to: Fazel S, Wolf A, Palm C, Lichtenstein P. Violent crime, suicide, and premature mortality in patients with schizophrenia and related disorders: a 38-year total population study in Sweden. *Lancet Psychiatry* 2014; **1:** 44–54.

Appendix table 1: Descriptive data for risk factors for unaffected siblings

	Men (n=13,577)	Women (n=12,780)
**SOCIOECONOMIC FACTORS**		
Income in lowest tertile	3,109 (22.9%)	3,262 (25.5%)
Born abroad	1,599 (11.8%)	1,581 (12.4%)
**PARENTAL FACTORS BEFORE MATCHING**		
Alcohol or drug use disorders	300 (2.2%)	295 (2.3%)
Any offence	663 (4.9%)	672 (5.3%)
Violent offence	246 (1.8%)	243 (1.9%)
Suicide	255 (1.9%)	225 (1.8%)
Psychiatric diagnosis	801 (5.9%)	810 (6.3%)
Born abroad	2,521 (18.6%)	2,458 (19.2%)
**INDIVIDUAL FACTORS BEFORE MATCHING**		
Alcohol use disorders	345 (2.5%)	144 (1.1%)
Drug use disorders	214 (1.6%)	106 (0.8%)
Alcohol offence	1,207 (8.9%)	115 (0.9%)
Drug offence	759 (5.6%)	151 (1.2%)
Alcohol or drug medication	24 (0.2%)	7 (0.1%)
Any offence	3,949 (29.1%)	1,059 (8.3%)
Violent offence	1,344 (9.9%)	167 (1.3%)
Non-violent offence	3,703 (27.3%)	998 (7.8%)
Self-harm	266 (2.0%)	422 (3.3%)
IQ* – high (7-9)	2,291 (21.9%)	NA
- medium (4-6)	5,224 (38.5%)	NA
- low (1-3)	2,945 (21.7%)	NA
Birth complications	282 (9.3%)	238 (8.4%)

Data are n (%), unless otherwise indicated. IQ=intelligence quotient. NA=not available. Data for birth complications were available for a sample of 10,540 men and 9,948 women. *IQ data were available for 10,460 men.

Appendix table 2: Descriptive data for risk factors for general population controls

	Men (n=292,420)	Women (n=193,520)
**SOCIOECONOMIC FACTORS**		
Income in lowest tertile	82,900 (28.3%)	58,471 (30.2%)
Born abroad	53,806 (18.4%)	35,626 (18.4%)
Single	174,325 (59.6%)	96,145 (49.7%)
**PARENTAL FACTORS BEFORE MATCHING**		
Alcohol or drug use disorders	9,744 (3.3%)	6,579 (3.4%)
Any offence	13,536 (4.6%)	8,931 (4.6%)
Violent offence	3,975 (1.4%)	2,574 (1.3%)
Suicide	3,138 (1.1%)	2,180 (1.1%)
Psychiatric diagnosis	12,996 (4.4%)	8,871 (4.6%)
Born abroad	21,104 (7.2%)	13,797 (7.1%)
**INDIVIDUAL FACTORS BEFORE MATCHING**		
Alcohol use disorders	2,834 (1.0%)	1,156 (0.6%)
Drug use disorders	1,570 (0.5%)	755 (0.4%)
Alcohol offence	2,809 (1.0%)	289 (0.1%)
Drug offence	2,670 (0.9%)	469 (0.2%)
Alcohol or drug medication	242 (0.1%)	78 (0.04%)
Any offence	54,633 (18.7%)	9,644 (5.0%)
Violent offence	14,452 (4.9%)	1,090 (0.6%)
Non-violent offence	50,775 (17.4%)	9,159 (4.7%)
Self-harm	3,133 (1.1%)	3,071 (1.6%)
IQ* – high (7-9)	49,682 (17.0%)	---
- medium (4-6)	113,339 (54.9%)	---
- low (1-3)	43,455 (21.0%)	---
Birth complications	6,365 (8.3%)	3,781 (8.5%)

Data are n (%), unless otherwise indicated. IQ=intelligence quotient. NA=not available. Data on birth complications were available for a sample of 76,427 men and 44,418 women. *IQ data were available for 206,476 men.

Appendix table 3: Risk factors for violent offence in patients with schizophrenia and related disorders, unaffected siblings, and general population controls

	**Schizophrenia and related disorders**	**Unaffected siblings**	**General population**
	**HR (95% CI)**	**HR (95% CI)**	**HR (95% CI)**
SOCIOECONOMIC FACTORS			
**Sex**			
- male	**3.3 (3.0-3.6)**	**6.3 (5.3-7.6)**	**7.8 (7.2-8.5)**
**Low income**			
- male	**1.4 (1.3-1.5)**	**1.6 (1.4-1.9)**	**1.7 (1.7-1.8)**
- female	**1.4 (1.2-1.7)**	**2.1 (1.5-3.0)**	**2.1 (1.8-2.5)**
**Born abroad**			
- male	**1.7 (1.5-1.8)**	**2.4 (2.0-2.8)**	**2.1 (2.0-2.2)**
- female	**1.4 (1.2-1.7)**	**1.8 (1.1-2.7)**	**2.0 (1.7-2.4)**
**Single**			
- male	1.0 (0.9-1.1)	n/a	1.0 (0.9-1.0)
- female	1.0 (0.8-1.2)	n/a	0.9 (0.8-1.1)
PARENTAL FACTORS BEFORE DIAGNOSIS/MATCHING			
**Alcohol or drug use disorders**			
- male	**1.8 (1.5-2.1)**	**1.6 (1.1-2.4)**	**2.5 (2.3-2.7)**
- female	**1.9 (1.4-2.7)**	**3.1 (1.5-6.2)**	**3.1 (2.4-4.0)**
**Any offence**			
- male	**1.3 (1.1-1.6)**	**1.4 (1.1-1.9)**	**1.7 (1.6-1.9)**
- female	**1.5 (1.1-2.0)**	1.7 (0.9-3.3)	**2.2 (1.7-2.9)**
**Violent offence**			
- male	**2.1 (1.8-2.6)**	**2.7 (1.9-3.8)**	**3.5 (3.1-3.9)**
- female	**2.0 (1.3-3.1)**	**3.9 (1.9-8.1)**	**4.7 (3.4-6.5)**
**Suicide**			
- male	1.3 (1.0-1.7)	**1.9 (1.3-2.8)**	**1.7 (1.4-2.0)**
- female	1.6 (1.0-2.6)	2.0 (0.7-5.3)	**1.8 (1.1-3.1)**
**Psychiatric diagnosis**			
- male	1.1 (1.0-1.3)	1.2 (0.9-1.6)	**1.6 (1.5-1.8)**
- female	**1.4 (1.1-1.9)**	**2.0 (1.2-3.5)**	**2.0 (1.5-2.6)**
**Born abroad**			
- male	**1.4 (1.3-1.6)**	**2.3 (2.0-2.6)**	**2.2 (2.0-2.3)**
- female	**1.3 (1.0-1.7)**	**2.0 (1.4-2.9)**	**1.8 (1.4-2.3)**
INDIVIDUAL FACTORS BEFORE DIAGNOSIS/MATCHING			
**Alcohol use disorders**			
- male	**2.6 (2.3-2.9)**	**7.4 (5.9-9.3)**	**9.0 (8.2-9.9)**
- female	**4.5 (3.4-6.0)**	**21.3 (12.7-35.6)**	**19.8 (14.6-26.7)**

**Drug use disorders**			
- male	**3.1 (2.8-3.5)**	**10.4 (8.1-13.4)**	**16.2 (14.6-17.9)**
- female	**5.1 (4.1-6.5)**	**23.4 (13.4-41.1)**	**36.0 (27.0-48.0)**
**Alcohol offence**			
- male	**2.8 (2.5-3.0)**	**5.2 (4.5-6.1)**	**16.7 (15.6-17.9)**
- female	**4.7 (3.3-6.7)**	**10.7 (5.6-20.5)**	**39.5 (27.4-56.8)**
**Drug offence**			
- male	**3.8 (3.5-4.1)**	**8.7 (7.4-10.2)**	**19.4 (18.1-20.8)**
- female	**6.2 (4.8-8.0)**	**28.6 (18.2-45.1)**	**51.6 (39.6-67.2)**
**Alcohol or drug medication**			
- male	1.2 (0.7-2.1)	3.7 (0.5-26.2)	**11.2 (7.1-17.9)**
- female	1.2 (0.2-8.8)	**---**	**---**
**Any offence**			
- male	**3.8 (3.5-4.1)**	**6.7 (5.8-7.7)**	**7.0 (6.7-7.3)**
- female	**5.0 (4.2-6.0)**	**12.7 (9.0-17.8)**	**13.9 (11.9-16.2)**
**Violent offence**			
- male	**4.3 (4.0-4.7)**	**9.9 (8.6-11.4)**	**11.6 (11.1-12.2)**
- female	**7.7 (6.2-9.6)**	**24.5 (15.5-38.6)**	**33.8 (27.0-42.2)**
**Non-violent offence**			
- male	**3.5 (3.2-3.8)**	**6.1 (5.3-7.0)**	**6.5 (6.3-6.8)**
- female	**4.9 (4.1-5.8)**	**12.2 (8.7-17.2)**	**12.8 (11.0-15.0)**
**Self-harm**			
- male	**1.8 (1.6-2.1)**	**5.8 (4.3-7.6)**	**5.7 (5.2-6.4)**
- female	**1.5 (1.2-1.9)**	**10.2 (6.8-15.5)**	**8.5 (6.6-10.9)**
**IQ (men only)**			
high (7-9)	**1.0 (ref)**	**1.0 (ref)**	**1.0 (ref)**
medium (4-6)	**1.4 (1.2-1.8)**	**2.3 (1.7-3.1)**	**2.9 (2.6-3.3)**
low (1-3)	**1.8 (1.5-2.2)**	**5.2 (3.8-7.0)**	**7.6 (6.8-8.4)**
**Birth complications**			
- male	1.0 (0.7-1.3)	**1.7 (1.0-2.8)**	1.0 (0.8-1.2)
- female	0.6 (0.3-1.6)	2.8 (0.9-8.3)	1.1 (0.6-2.1)

Hazard ratios (HR) and 95% confidence intervals (CI) significant to p<0.05 level are in **bold**.
Adjusted for age at diagnosis.

Appendix table 4: Risk factors for suicide in patients with schizophrenia and related disorders, unaffected siblings, and general population controls

	Schizophrenia and related disorders	Unaffected siblings	General population
	HR (95% CI)	HR (95% CI)	HR (95% CI)
**SOCIOECONOMIC FACTORS**			
**Sex**			
- male	**1.5 (1.3-1.8)**	**2.1 (1.5-2.9)**	**2.8 (2.3-3.3)**
**Low income**			
- male	1.0 (0.9-1.2)	**1.7 (1.2-2.5)**	**1.4 (1.2-1.6)**
- female	1.3 (1.0-1.7)	1.3 (0.8-2.3)	**1.6 (1.1-2.1)**
**Born abroad**			
- male	**0.8 (0.6-0.9)**	0.5 (0.2-1.1)	**0.6 (0.4-0.8)**
- female	0.8 (0.6-1.1)	0.4 (0.1-1.4)	1.2 (0.8-1.9)
**single**			
- male	**0.5 (0.4-0.6)**	n/a	1.0 (0.8-1.1)
- female	**0.6 (0.5-0.8)**	n/a	1.0 (0.7-1.3)
**PARENTAL FACTORS BEFORE DIAGNOSIS/MATCHING**			
**Alcohol or drug use disorders**			
- male	1.2 (0.9-1.7)	2.1 (0.9-5.3)	**2.4 (1.8-3.2)**
- female	1.2 (0.7-2.1)	0.9 (0.1-6.3)	**3.9 (2.4-6.4)**
**Any offence**			
- male	0.8 (0.6-1.2)	1.2 (0.5-2.8)	**1.6 (1.2-2.1)**
- female	1.0 (0.6-1.7)	1.3 (0.4-4.3)	**2.0 (1.2-3.6)**
**Violent offence**			
- male	**0.4 (0.2-0.9)**	1.1 (0.3-4.5)	**2.2 (1.3-3.5)**
- female	0.6 (0.2-1.6)	---	**3.0 (1.2-7.4)**
**Suicide**			
- male	**1.8 (1.1-2.7)**	2.4 (1.0-5.9)	**2.5 (1.6-3.9)**
- female	**1.9 (1.0-3.6)**	2.2 (0.5-8.9)	**3.1 (1.4-7.1)**
**Psychiatric diagnosis**			
- male	1.0 (0.7-1.3)	1.5 (0.8-2.9)	**2.4 (1.8-3.1)**
- female	1.4 (1.0-2.1)	---	**2.1 (1.2-3.7)**
**Born abroad**			
- male	1.0 (0.8-1.3)	1.2 (0.7-1.9)	1.3 (0.9-1.7)
- female	0.7 (0.5-1.1)	1.1 (0.6-2.2)	**2.0 (1.2-3.3)**
**INDIVIDUAL FACTORS BEFORE DIAGNOSIS/MATCHING**			
**Alcohol use disorders**			
- male	**1.5 (1.2-2.1)**	**9.1 (5.4-15.2)**	**8.1 (5.8-11.4)**
- female	**2.3 (1.4-3.8)**	**8.0 (2.5-25.7)**	**15.9 (7.8-32.5)**

**Drug use disorders**			
- male	**1.7 (1.3-2.1)**	**20.3 (12.7-32.4)**	**17.3 (12.6-23.7)**
- female	**1.8 (1.2-2.8)**	3.3 (0.5-24.1)	**24.0 (12.2-47.3)**
**Alcohol offence**			
- male	**1.5 (1.2-1.9)**	**3.6 (2.4-5.4)**	**10.5 (8.2-13.4)**
- female	1.4 (0.6-3.2)	2.0 (0.3-14.4)	**8.4 (2.1-33.9)**
**Drug offence**			
- male	**1.3 (1.0-1.6)**	**8.3 (5.6-12.4)**	**11.5 (8.9-14.8)**
- female	1.6 (0.9-2.8)	**5.6 (1.7-18.0)**	**25.6 (13.0-50.2)**
**Alcohol or drug medication**			
- male	1.3 (0.4-4.2)	32.1 (4.3-241.5)	**8.4 (1.2-60.1)**
- female	**7.7 (2.4-24.4)**	---	**80.9 (10.9-598.4)**
**Any offence**			
- male	**1.5 (1.3-1.8)**	**3.1 (2.2-4.5)**	**3.6 (3.1-4.2)**
- female	**1.6 (1.1-2.1)**	**2.3 (1.1-4.9)**	**4.6 (3.1-6.9)**
**Violent offence**			
- male	**1.5 (1.2-1.8)**	**3.8 (2.6-5.8)**	**4.2 (3.4-5.1)**
- female	1.2 (0.7-2.1)	1.9 (0.3-13.4)	**10.9 (5.4-22.3)**
**Non-violent offence**			
- male	**1.5 (1.3-1.8)**	**3.4 (2.4-4.9)**	**3.8 (3.3-4.5)**
- female	**1.5 (1.1-2.1)**	**2.5 (1.2-5.2)**	**4.4 (2.9-6.7)**
**Self-harm**			
- male	**2.2 (1.8-2.7)**	**9.3 (5.3-16.5)**	**7.1 (5.2-9.8)**
- female	**2.3 (1.7-3.0)**	**9.7 (5.1-18.5)**	**13.4 (8.7-20.7)**
**IQ (men only)**			
high (7-9)	1.0 (ref)	**1.0 (ref)**	**1.0 (ref)**
medium (4-6)	0.9 (0.7-1.2)	1.2 (0.7-2.3)	**1.5 (1.1-1.9)**
low (1-3)	0.8 (0.6-1.0)	**2.0 (1.1-3.8)**	**2.6 (2.0-3.4)**
**Birth complications**			
- male	1.2 (0.7-2.2)	3.2 (0.9-11.8)	1.3 (0.7-2.5)
- female	1.4 (0.6-3.2)	---	---

Hazard ratios (HR) and 95% confidence intervals (CI) significant to p<0.05 level are in **bold**. Adjusted for age at diagnosis.

Appendix table 5: Risk factors for premature mortality in patients with schizophrenia and related disorders, unaffected siblings, and general population controls

	Schizophrenia and related disorders	Unaffected siblings	General population
	**HR (95% CI)**	**HR (95% CI)**	**HR (95% CI)**
**SOCIOECONOMIC FACTORS**			
**Sex**			
- male	**1.6 (1.4-1.8)**	**2.0 (1.7-2.4)**	**1.7 (1.6-1.8)**
**Low income**			
- male	1.1 (1.0-1.2)	**1.3 (1.1-1.6)**	**1.3 (1.2-1.4)**
- female	**1.3 (1.1-1.5)**	1.2 (0.9-1.6)	**1.2 (1.1-1.3)**
**Born abroad**			
- male	**0.9 (0.7-1.0)**	1.1 (0.8-1.5)	1.1 (0.9-1.2)
- female	0.8 (0.6-1.0)	0.7 (0.4-1.2)	**1.3 (1.1-1.5)**
**single**			
- male	**0.6 (0.6-0.7)**	n/a	0.9 (0.9-1.0)
- female	**0.7 (0.6-0.9)**	n/a	1.1 (1.0-1.2)
**PARENTAL FACTORS BEFORE DIAGNOSIS/MATCHING**			
**Alcohol or drug use disorders**			
- male	1.3 (1.0-1.6)	1.5 (0.8-2.7)	**1.9 (1.6-2.2)**
- female	1.3 (0.8-1.9)	1.4 (0.6-3.3)	**1.4 (1.0-1.8)**
**Any offence**			
- male	0.9 (0.7-1.2)	1.1 (0.6-1.7)	**1.4 (1.2-1.7)**
- female	1.1 (0.8-1.6)	1.0 (0.5-2.1)	1.2 (0.9-1.6)
**Violent offence**			
- male	**0.4 (0.2-0.7)**	1.4 (0.7-3.1)	**2.1 (1.6-2.6)**
- female	0.6 (0.3-1.3)	0.5 (0.1-3.3)	1.1 (0.6-1.9)
**Suicide**			
- male	1.4 (1.0-2.0)	**1.5 (0.8-2.7)**	**1.7 (1.3-2.2)**
- female	**1.7 (1.0-2.8)**	0.9 (0.3-2.8)	1.2 (0.8-1.9)
**Psychiatric diagnosis**			
- male	1.0 (0.8-1.3)	1.6 (1.1-2.3)	**1.6 (1.4-1.9)**
- female	1.3 (0.9-1.7)	0.7 (0.3-1.5)	1.0 (0.7-1.3)
**Born abroad**			
- male	1.1 (0.9-1.3)	**1.4 (1.1-1.8)**	**1.4 (1.3-1.7)**
- female	0.9 (0.7-1.3)	0.9 (0.6-1.4)	**1.4 (1.1-1.8)**
**INDIVIDUAL FACTORS BEFORE DIAGNOSIS/MATCHING**			
**Alcohol use disorders**			
- male	**1.9 (1.6-2.4)**	**6.4 (4.7-8.6)**	**7.7 (6.6-9.1)**
- female	**2.4 (1.7-3.4)**	**8.6 (4.5-16.3)**	**9.9 (7.1-13.7)**

**Drug use disorders**			
- male	**1.9 (1.6-2.3)**	**11.8 (8.7-16.0)**	**13.9 (11.8-16.4)**
- female	**2.5 (1.9-3.4)**	**4.1 (1.5-11.1)**	**9.9 (6.9-14.2)**
**Alcohol offence**			
- male	**1.7 (1.5-2.0)**	**3.1 (2.5-3.9)**	**8.2 (7.2-9.3)**
- female	1.6 (0.9-2.8)	2.4 (0.9-6.4)	**6.4 (3.7-11.1)**
**Drug offence**			
- male	**1.8 (1.5-2.0)**	**5.4 (4.3-6.9)**	**10.9 (9.6-12.3)**
- female	**2.1 (1.5-3.1)**	**4.1 (2.0-8.4)**	**10.9 (7.6-15.5)**
**Alcohol or drug medication**			
- male	0.8 (0.3-2.4)	7.7 (1.1-56.0)	**6.4 (2.4-17.2)**
- female	**4.5 (1.4-14.2)**	**---**	**14.7 (3.6-59.0)**
**Any offence**			
- male	**1.7 (1.5-1.9)**	**2.6 (2.2-3.2)**	**2.9 (2.7-3.1)**
- female	**1.7 (1.3-2.1)**	**3.1 (2.1-4.5)**	**2.7 (2.2-3.2)**
**Violent offence**			
- male	**1.6 (1.4-1.8)**	**3.8 (3.0-4.7)**	**3.9 (3.6-4.3)**
- female	**1.8 (1.3-2.6)**	**4.8 (2.3-9.7)**	**4.2 (2.8-6.3)**
**Non-violent offence**			
- male	**1.7 (1.5-1.9)**	**2.7 (2.3-3.3)**	**3.0 (2.8-3.2)**
- female	**1.7 (1.3-2.1)**	**2.9 (1.9-4.3)**	**2.7 (2.3-3.3)**
**Self-harm**			
- male	**1.9 (1.6-2.2)**	**5.5 (3.8-7.9)**	**4.2 (3.4-5.0)**
- female	**2.1 (1.7-2.6)**	**5.3 (3.4-8.1)**	**4.3 (3.3-5.5)**
**IQ (men only)**			
high (7-9)	1.0 (ref)	**1.0 (ref)**	**1.0 (ref)**
medium (4-6)	1.1 (0.8-1.3)	1.4 (1.0-2.1)	**1.4 (1.2-1.6)**
low (1-3)	1.0 (0.8-1.2)	**2.4 (1.7-3.5)**	**2.3 (2.0-2.7)**
**Birth complications**			
- male	**1.9 (1.2-2.9)**	**6.1 (3.0-12.6)**	1.2 (0.8-1.7)
- female	1.3 (0.6-2.8)	**---**	1.0 (0.5-2.1)

Hazard ratios (HR) and 95% confidence intervals (CI) significant to p<0.05 level are in **bold**.
Adjusted for age at diagnosis.

Appendix table 6: Adjusted odds ratios of adverse outcomes in patients with schizophrenia and related disorders compared with general population controls, by age group.

Age at diagnosis	Violent offence aOR (95% CI)	Suicide aOR (95% CI)	Premature mortality aOR (95% CI)
**<25 (n=7,965)**	5.9 (5.5-6.3)	19.8 (16.9-23.1)	8.1 (7.2-9.0)
**25-34 (n=10,155)**	8.7 (8.1-9.3)	22.2 (19.2-25.8)	8.9 (8.1-9.8)
**35-44 (n=5153)**	9.4 (8.2-10.7)	19.0 (14.2-25.4)	6.5 (5.6-7.6)
**45-54 (n=1,024)**	6.0 (3.6-10.1)	13.8 (3.9-49.5)	4.4 (2.7-7.3)

General population controls are matched by age and sex. aOR = odds ratios, adjusted for low family income and being born abroad.

Appendix table 7: Adjusted odds ratios of adverse outcomes in patients with schizophrenia and related disorders compared with general population controls, by year of diagnosis

	First diagnosis before 2001 aOR (95% CI)	First diagnosis in or after 2001 aOR (95% CI)	Statistical difference between aORs
**Violence**	7.3 (6.9-7.7)	7.9 (7.3-8.7)	p=0.115
**Suicide**	20.2 (18.0-22.6)	23.2 (18.5-29.2)	p=0.275
**Premature mortality**	8.2 (7.7-8.8)	7.6 (6.6-8.7)	p=0.210

General population controls are matched by age and sex. aOR = odds ratios, adjusted for low family income and being born abroad.

Appendix table 8: Adjusted odds ratios of adverse outcomes in patients with schizophrenia and related disorders compared with general population controls, by diagnostic group (ICD-9 and ICD-10 only)

	Schizophrenia aOR (95% CI)	Other non-affective psychoses aOR (95% CI)	Statistical difference between aORs
**Violence**	7.1 (6.7-7.6)	7.7 (7.2-8.2)	p=0.083
**Suicide**	21.3 (17.8-25.5)	22.0 (19.2-25.2)	p=0.776
**Premature mortality**	8.3 (7.4-9.3)	8.2 (7.5-8.9)	p=0.858

General population controls are matched by age and sex. aOR = odds ratios, adjusted for low family income and being born abroad.

Appendix table 9: Effects of year of diagnosis and annual inpatient nights on ratio of odds ratios of adverse outcomes

**% increases in ratio of odds ratios, *by calendar year***		
	Unadjusted (as in paper)	Substance abuse adjusted
Violence	1.1% (0.1% to 2.2%)	1.0% (-0.2% to 2.1%)
Suicide	1.7% (-0.9% to 4.3%)	1.7% (-1.0 to 4.4%)
Premature mortality	0.2% (-1.3% to 1.6%)	0.3% (-1.2% to 1.8%)
**% increases in ratio of odds ratios, *by 1,000,000 fewer inpatient nights per year***		
	Unadjusted (as in paper)	Substance abuse adjusted
Violence	5.6% (2.6% to 8.4%)	5.0% (1.9% to 7.9%)
Suicide	5.5% (-1.3% to 11.8%)	5.6% (-1.4% to 12.1%)
Premature mortality	3.4% (-0.6% to 7.2%)	3.6% (-0.4 to 7.5%)

The ratio of odds ratios measures changes in rates of adverse outcomes in patients with schizophrenia and related disorders compared with unaffected siblings.
